# Praeruptorin A Inhibits Human Cervical Cancer Cell Growth and Invasion by Suppressing MMP-2 Expression and ERK1/2 Signaling

**DOI:** 10.3390/ijms19010010

**Published:** 2017-12-21

**Authors:** Min-Hua Wu, Chia-Liang Lin, Hui-Ling Chiou, Shun-Fa Yang, Ching-Yi Lin, Chung-Jung Liu, Yi-Hsien Hsieh

**Affiliations:** 1 Institute of Medicine, Chung Shan Medical University, Taichung 40201, Taiwan; 3150@ccgh.com.tw (M.-H.W.); ysf@csmu.edu.tw (S.-F.Y.); 2 Department of Laboratory, Chung-Kang Branch, Cheng-Ching General Hospital, Taichung 40764, Taiwan; 3 Institute of Biochemistry, Microbiology and Immunology, Chung Shan Medical University, Taichung 40201, Taiwan; hiking0210001@gmail.com; 4 School of Medical Laboratory and Biotechnology, Chung Shan Medical University, Taichung 40201, Taiwan; hlchiou@csmu.edu.tw; 5 Department of Medical Research, Chung Shan Medical University Hospital, Taichung 40201, Taiwan; 6 Division of Chest Medicine, Department of Internal Medicine, Taichung Veterans General Hospital, Taichung 40201, Taiwan; chingyii@vghtc.gov.tw; 7 Division of Gastroenterology, Department of Internal Medicine, Kaohsiung Medical University Hospital, Kaohsiung 80708, Taiwan; 8 Center for Stem Cell Research, Kaohsiung Medical University, Kaohsiung 80708, Taiwan; 9 Department of Biochemistry, School of Medicine, Chung Shan Medical University, Taichung 40201, Taiwan; 10 Clinical laboratory, Chung Shan Medical University Hospital, Taichung 40201, Taiwan

**Keywords:** Praeruptorin A, cervical cancer cells, ERK1/2, invasion, MMP-2, TIMP-2

## Abstract

Praeruptorin A (PA) is a pyranocumarin present in the dried root of Peucedanumpraeruptorum Dunn that has anticancer effects against several types of cells. However, the effect of PA on human cervical cancer cells is unknown. Our results indicate that PA significantly inhibited cell proliferation, colony formation, migration, invasion, and wound closure of HeLa and SiHa cells, induced cell cycle arrest at G0/G1 phase, upregulated Rb, p16, p21 and p27 proteins and downregulated cyclin D1 and S-phase kinase-associated protein 2 (Skp2) proteins. PA also significantly reduced expression of matrix metalloproteinase-2 (MMP-2) and increased expression of tissue inhibitor of metalloproteinase-2 (TIMP-2). In addition, PA suppressed ERK1/2 activation and increased the effect of PD98059 (a specific MEK1/2 inhibitor) in downregulation of MMP-2 and upregulation of TIMP-2. PA treatment inhibited the effect of 12-*O*-tetradecanoylphorbol-13-acetate (TPA) on upregulation of ERK1/2 activation, MMP-2 expression, cellular migration, and invasion of HeLa cells. Our findings are the first to demonstrate the activity of PA against cervical cancer cells, and suggest this agent has promise as a therapeutic agent in treatment of human cervical cancer.

## 1. Introduction

Cervical cancer is the second most common gynecologic cancer worldwide, and there were 530,000 new diagnoses in 2012 and 300,000 deaths in 2015. Cervical cancer patients mainly die from disease recurrence or progression, despite the use of advanced chemotherapeutic treatments [1]. The high lethality is due to this cancer’s resistance to available chemotherapy agents, its unusual ability to adapt to radiotherapy [2], and the lack of alternative treatment options. Metastasis of cervical cancer to other sites, such as the lymph nodes, lung, bone, liver, and bowel, are critical factors that contribute to its high mortality rate [3,4]. So far, surgical resection is the only effective therapeutic method for cervical cancer patients following unsuccessful chemotherapy. Therefore, there is an urgent need for novel treatments that inhibit the growth and metastasis of cervical cancer.

Metastasis is the major cause of poor clinical outcomes and high mortality in cervical cancer. Metastasis is a complex process that includes alterations in cell adhesion, migration, invasion, and proteolytic degradation of the extracellular matrix (ECM). ECM degradation by extracellular proteinases contributes to tumor progression, invasion, and metastasis [5]. Matrix metalloproteinases (MMPs) are a group of proteinases that are primarily responsible for ECM degradation in vivo [6]. These are zinc-containing enzymes that include interstitial collagenases, gelatinases, stromelysin, matrilysin, metalloelastase, and membrane-type MMPs [7]. MMP-2 and MMP-9 have important roles in the development of malignant cervical cancer in animal models and humans [8,9]. Several endogenous inhibitors, such as tissue inhibitor of metalloproteinase (TIMPs), regulate MMP through modification of its activity and stability [10]. 

**Figure 1:**
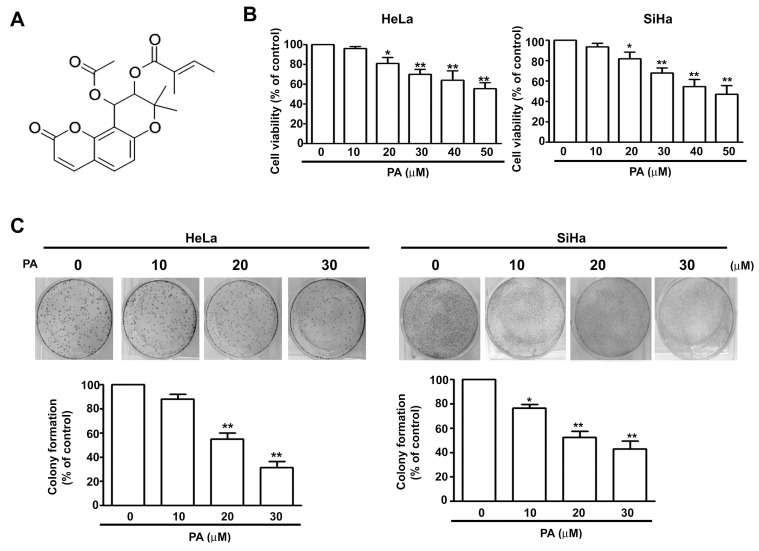
Effect of Praeruptorin A (PA) on viability of human cervical cancer cells. (**A**) Structure of Praeruptorin A (PA); (**B**) viability of HeLa and SiHa cells treated with various concentrations of PA (0 to 50 μM) for 24 h, based on the 3-(4,5-Dimethylthiazol-2-yl)-2,5-diphenyltetrazolium bromide (MTT) assay; (**C**) colony formation of HeLa and SiHa cells treated with various concentrations of PA (0 to 30 μM), with staining by crystal violet. Here and below, values are means and standard errors of 3 replicates, * indicates *p* < 0.05 versus control, and ** indicates *p* < 0.01 versus control.

Many researchers use screening programs to examine numerous plant compounds for their potential efficacy in the treatment of many different cancers. Herbal medicines and natural foods might have synergistic effects with conventional antitumor agents, or might even be suitable replacements for conventional chemotherapy agents [11]. Praeruptorin A (PA, Figure 1A) is a major bioactive pyranocoumarin that occurs in the dried root of Peucedanumpraeruptorum Dunn, a plant in the carrot family (Apiaceae) [12]. Many pharmacological studies indicate that an extract of P. praeruptorum might have beneficial effects, due to its anti-inflammation [13], neuroprotection [14] and anti-cancer effect [15]. PA is reported to induce apoptosis in human multidrug resistant (MDR) KB-V1 cells [16]. However, the effects and molecular mechanisms of PA on the proliferation and metastasis of human cervical cancer cells are unknown. 

It is necessary to identify and characterize potentially effective plant-based products against human cervical cancer cells. Thus, we investigated the effect of PA on the growth, migration, and invasion, of human cervical cancer (HeLa and SiHa) cells and the molecular mechanisms of these effects. The results demonstrate that PA inhibited growth, induced cell cycle arrest at G0/G1 phase, increased the levels of p16, p21, p27 and Rb levels, and reduced the levels of cyclin D1 and Skp2. PA also significantly suppressed the induction of cell invasion and motility induced by 12-*O*-tetradecanoylphorbol-13-acetate (TPA, a tumor promoter) through inactivation of ERK1/2 signaling, downregulation of MMP-2, and upregulation of TIMP-2 in HeLa cells. Our results suggest that PA inhibits the proliferation and invasion of human cervical cancer cells by disruption of ERK1/2 signaling.

## 2. Results

### 2.1. PA Reduces Viability and Colony Formation of HeLa and SiHa Cells

Figure 1A shows the chemical structure of PA. We initially tested the effect of PA on cell viability and colony formation in HeLa cells and SiHacells by addition of various concentrations of PA for 24 h, followed by the 3-(4,5-Dimethylthiazol-2-yl)-2,5-diphenyltetrazolium bromide (MTT) assay and assessment of colony formation. The results showed that PA significantly reduced cell viability (Figure 1B) and colony formation (Figure 1C) in both cell lines in a concentration-dependentmanner.

### 2.2. PA Induces Cell Cycle Arrest at G0/G1 Phase in HeLa Cells

**Figure 2:**
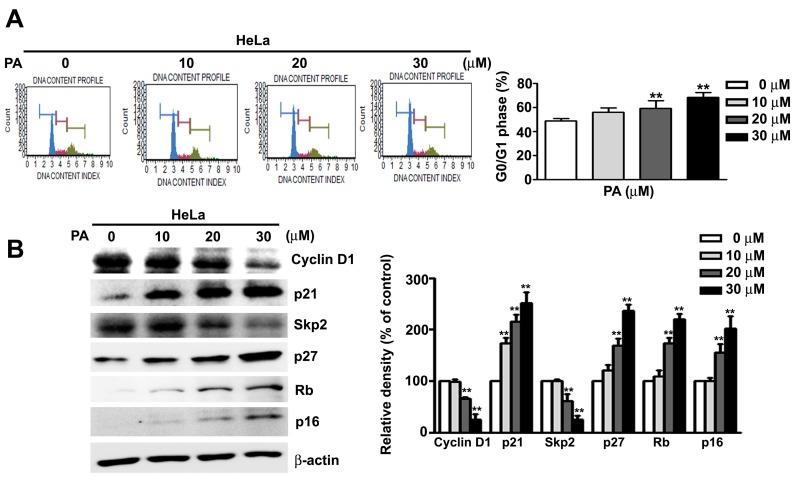
Effect of PA on cell cycle regulation in HeLa cells. (**A**) Cells were treated with various concentrations of PA (0 to 30 μM) and cell cycle progression was measured by flow cytometry; (**B**) expression of proteins that regulate the cell cycle were measured after treatment of cells with various concentrations of PA (0 to 30 μM) for 24 h. Values are means and standard errors of 3 replicates, ** indicates *p* < 0.01 versus control.

We used flow cytometry to further examine the effect of PA on regulation of the cell cycle in HeLa cells. The results showed that PA induced cell cycle arrest at the G0/G1 phase in a concentration-dependent manner (Figure 2A). Moreover, western blotting of proteins with critical roles in cell cycle regulation indicated that PA treatment led to a concentration-dependent reductions in the levels of cyclin D1 and Skp2, but increased levels of p21, p27, Rb, and p16 (Figure 2B). Our results confirmed that PA inhibit cell proliferation, accompany with G0/G1 phase arrest in HeLa cells.

### 2.3. PA Inhibits Cell Migration/Invasion and MMP-2 Expression in HeLa and SiHa Cells

**Figure 3:**
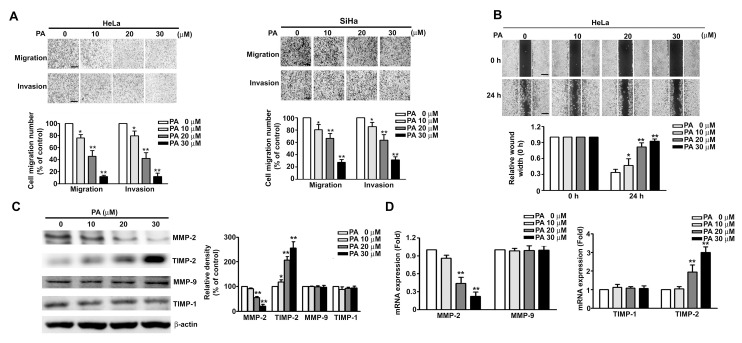
Effect of PA on cell migration/invasion, wound closure, and protein expression of MMPs and TIMPs in SiHa and HeLa cells. (**A**,**B**) Cells were treated with various concentrations of PA (0 to 30 μM) for 24 h, followed by measurement of cell migration and invasion and relative wound width. (**C**,**D**) Cells were treated as above, then harvested for measurement of MMP-2, MMP-9, TIMP-1, TIMP-2 proteins and mRNAs by western blotting and RT-qPCR. Values are means and standard errors of 3 replicates. ** *p* < 0.01 versus control; * *p* < 0.01 versus only PA treatment. Scale bar, 50 μm.

We also found that PA inhibited the migration and invasion of HeLa and SiHa cells in a concentration-dependent manner (Figure 3A). Similarly, use of the wound-healing assay indicated that the rate of wound closure also increased in a concentration-dependent manner in HeLa cells treated with PA (Figure 3B). MMP-2 and MMP-9 have been reported to play a critical role in cancer cell migration and invasion by contributing to the degradation of the ECM and cancer progression [17]. We next measured the effect of PA on mRNA and protein expression of MMP-2, MMP-9, TIMP-1, and TIMP-2 (which have critical roles in cancer cell migration and invasion) in HeLa cells by use of western blotting and RT-qPCR. The results showed that the protein and mRNA levels of MMP-2 were significantly reduced and those of TIMP-2 were significantly elevated at PA concentrations of 20 and 30 μM (Figure 3C,D). However, PA had no effect on the protein or mRNA levels of MMP-9 or TMP-1 at the tested concentrations. 

**Figure 4:**
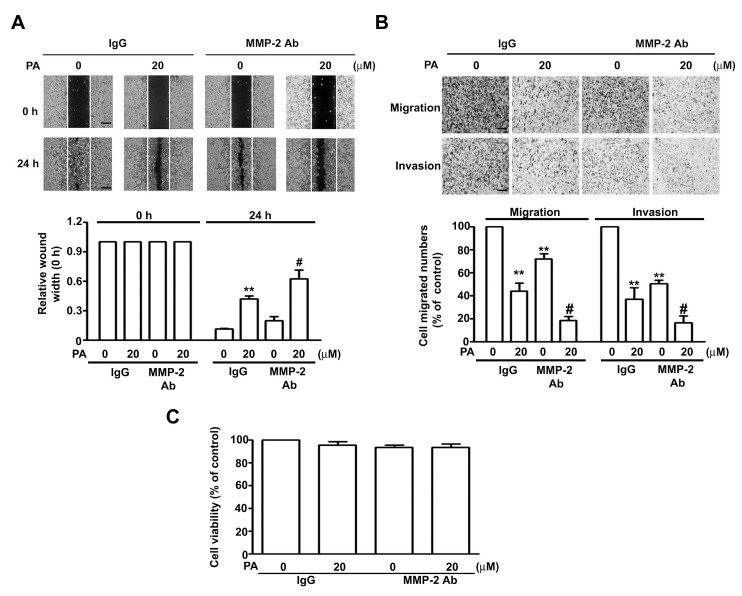
Effect of MMP-2 protein on PA inhibits cell migration and invasion in human HeLa cells. (**A**) HeLa cells were treated with/without 1 μg/mL of IgG or anti-MMP-2 neutralizing antibody (MMP-2 Ab) in the presence or absence of PA (20 μM) for 24 h, then followed by measuring the capacity of cell migration and invasion; (**B**) the wound-healing assay was expressed as relative wound width; (**C**) cell viability was measured by MTT assay. Values are means and standard errors of 3 replicates. ** *p* < 0.01 versus control; # *p* < 0.01 versus only PA treatment. Scale bar, 50 μm.

To further confirm the role of MMP-2 in inhibiting PA effects, we demonstrate that MMP-2 inhibition with an antibody (MMP-2 Ab) against MMP-2 decreased the mobility (Figure 4A), migration and invasion (Figure 4B) of HeLa cells compared with goat IgG antibody (as a control), and these effects were more marked in cells co-treated with both PA and MMP-2 antibody (Figure 4A,B), but no affect in cell growth (Figure 4C). Thus, these results confirm the role of MMP-2 in PA-inhibited cell migration and invasion in HeLa cells.

### 2.4. Role of ERK1/2 in PA-Modified MMP-2 and TIMP-2 Expression in HeLa Cells

**Figure 5:**
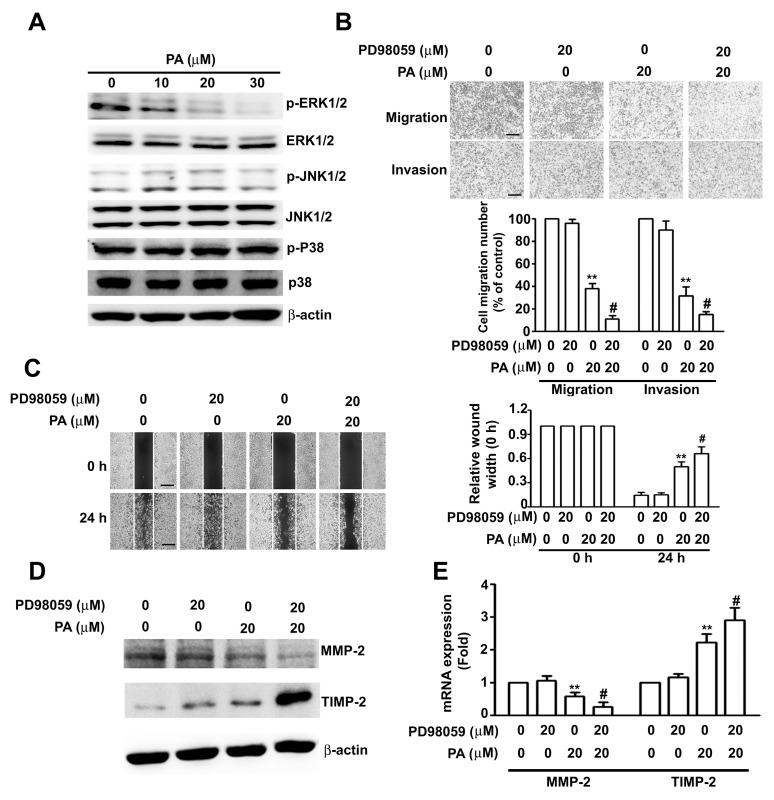
Role ERK1/2 in PA-regulated MMP-2 and TIMP-2 expression in HeLa cells. (**A**) Cells were treated with various concentrations of PA (0 to 30 μM) for 24 h, then harvested and lysed for measurement target proteins by western blotting; (**B**) cells were treated with or without PA in the absence or presence of PD98059 (specific MEK1/2 inhibitor) for 24 h, followed by measurement of migration and invasion; (**C**) cells were treated as above for 24 h, followed by measurement of relative wound width; (**D**,**E**) Protein and mRNA expression of MMP-2 and TIMP-2 were measured by western blotting assay and RT-qPCR. Values are means and standard errors of 3 replicates. ** *p* < 0.01 versus control; # *p* < 0.01 versus only PA treatment. Scale bar, 50 μm.

We next sought to identify the signal transduction pathway(s) involved in PA-reduced migration and invasion in HeLa cells. Thus, we treated HeLa cells with different concentrations of PA and then performed western blotting for proteins that have critical roles in signaling pathways involved in cancer. HeLa cells were treated with different concentration of PA (0, 10, 20 and 30 μM) for 24 h, the result show that PA inhibited the phosphorylation of ERK1/2, but had no effect on the phosphorylation of JNK1/2 and p38 (Figure 5A). We further examined the role of the ERK1/2 signaling pathway in PA by treatment of HeLa cells with PD98059 (a specific MEK1/2 inhibitor) to specifically block the expression ERK1/2. The results show that PD98059 significantly enhanced the effects of PA on inhibition of migration, invasion (Figure 5B), and wound closure (Figure 5C), on downregulation of MMP-2 protein and mRNA, and on upregulation of TIMP-2 protein and mRNA (Figure 5D,E). These results suggest that ERK1/2 signaling pathway(s) have a role in PA-induced inhibition of migration and invasion of HeLa cells.

### 2.5. PA Inhibits TPA-Induced ERK1/2 Activation, MMP-2 Expression, and Migration/Invasion in HeLa Cells

**Figure 6:**
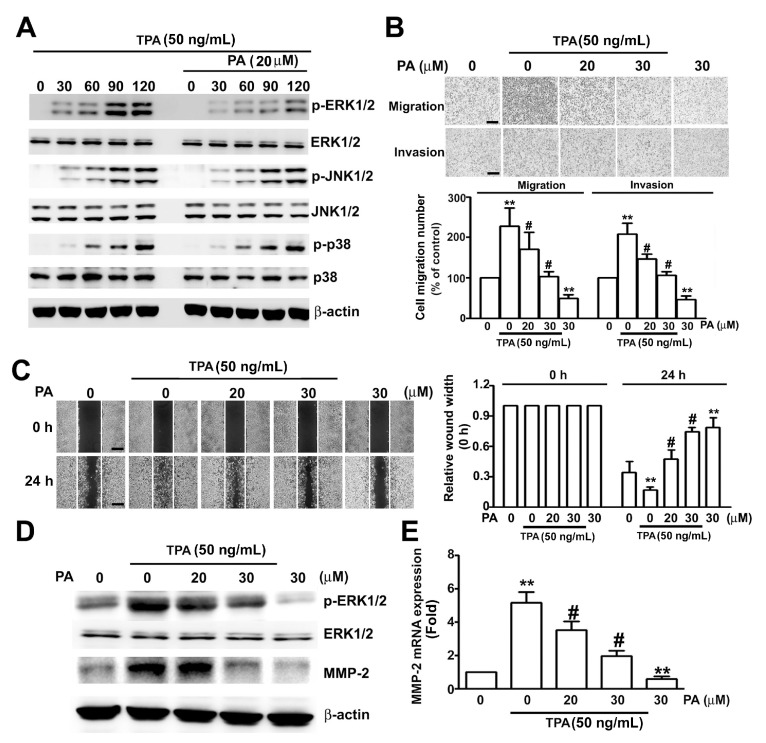
Effect of PA on TPA-induced ERK1/2 activation, MMP-2 expression, and migration/invasion of HeLa cells. (**A**) Cells were pretreated with or without PA (20 μM) for 2 h, then added TPA (50 ng/mL) for different times (0, 30, 60, 90 and 120 min), then harvested and lysed at the indicated times for measurement of target proteins by western blotting; (**B**) cells were pretreated with PA (20 to 30 μM) for 2 h, then in the absence or presence of TPA (50 ng/mL) for 24 h, followed by measurement of migration and invasion; (**C**) cells were treated as above for 24 h, followed by measurement of relative wound width; (**D**,**E**) cells were pretreated with PA (20 to 30 μM) for 2 h, then in the presence or absence of TPA (50 ng/mL) for 24 h, followed by measurement of ERK1/2 phosphorylation and MMP-2 protein and mRNA levels by western blotting and RT-qPCR assay. Values are means and standard errors of 3 replicates. ** *p* < 0.01 versus control; # *p* < 0.01 versus only TPA treatment. Scale bar, 50 μm.

We also examined the effect of PA (20 μM) on phosphorylation of MAPKs in HeLa cells induced by TPA (50 ng/mL) for different times (30, 60, 90 and 120 min). Treatment with TPA alone led to time-dependent phosphorylation of ERK1/2, JNK1/2, and p38 proteins, however, only TPA-induced ERK1/2 phosphorylation was reduced by PA (20 µM) treatment (Figure 6A). We furtherexamined whether PA inhibited the TPA-induced migration and invasion of HeLa cells, pretreatment of HeLa cells with PA (20 and 30 μM) in the presence or absence of TPA (50 ng/mL) for 24 h. These results showed that PA significantly inhibited the TPA-induced cell migration, invasion (Figure 6B), and wound closure (Figure 6C).To confirm whether PA-inhibited TPA-induced phosphorylation of ERK1/2 and MMP-2 expression in HeLa cells, we also found thatPA inhibited the TPA-induced phosphorylation of ERK1/2, and expression of MMP-2 protein and mRNA in HeLa cells (Figure 6D,E). Thus, PA reversed the effect of TPA on HeLa cells.

## 3. Discussion

Many plant-derived compounds have anti-cancer activities. Previous studies of prenylated coumarins indicated that ethanol extracts of P. praeruptorum exhibit in vitro cytotoxic activity against several human cancer cell lines [18]. Additional research reported that an angular pyranocoumarin extracted from P. praeruptoruon inhibited the proliferation and induced the apoptosis of U266 cells by upregulating caspase-8 and -3 proteins, and downregulating phospho-ERK and phospho-AKT proteins and hTERT mRNA [19]. Pyranocoumarins from root extracts of P. praeruptorum also downregulated nitric oxide (NO) production, and inhibited the efflux of drugs mediated by multidrug-resistance (MDR) proteins [16]. PA, isolated from the roots of P. praeruptorum, has antiproliferative and cytotoxic effects on human gastric cancer (SGC7901) cells and enhances the inhibitory effects of doxorubincin (DOX) in these cells [15]. We investigated the effects and underlying mechanism of PA on the proliferation of HeLa cells. Our results indicate that PA inhibited cell proliferation and led to cell cycle arrest at the G0/G1 phase. This effect appears to be due to its downregulation of cyclin D1 and Skp2 proteins, and upregulation of p16, p21, p27 and Rb proteins. More generally, our findings suggest that PA appears to slow the progression of human cervical cancer by modulating the cell cycle. In our study, we found that PA could not inhibit cell viability in IgG-treated cells (Figure 4C), we speculated on possible mechanism that PA bind the Fc portion of IgG interfering with PA inhibitory effect on HeLa cells. These results are similar to those of a previousstudy, which showed thatquercetin inhibit immunoglobulin secretion of IgG, IgM, and IgA isotypes in vitro [20]. However, more studies are needed to clarify the effect of IgG interference with PA in HeLa cells.

Remodeling of the ECM contributes to migration and invasion of cancer cells during distant metastasis. In particular, disrupting the interaction between cells and the ECM occurs as malignancy progresses [21]. Upregulation or activation of MMPs is associated with ECM remodeling, tumor cell invasion, and metastasis [22,23]. Thus, increased expression of MMP-2 and MMP-9, and decreased expression of TIMP-1 and TIMP-2 are potential markers of the invasive and metastatic potential of the squamous cervical carcinoma (SCC) [24]. Staurosporine (a kinase inhibitor) appears to induce an anti-tumor response in the cervical tumor microenvironment, and inhibits cancer progression and metastases through suppression of MMP-1 and MMP-2 [25]. Human papillomavirus (HPV) 16-E6 and -E7 are oncoproteins that together promote cervical cancer invasiveness by specifically increasing MMP-2 transcription [26]. The anti-viral drugs ribavirin and indinavir appear to protect against HPV-18-induced cervical cancer by decreasing the secretion of MMP-2 and MMP-9 [27]. In addition, MMP-2 and MMP-9 expression are associated with the progression of cervical cancer when exposed to low concentrations of arsenic trioxide and humic acid, and both of these proteins play important roles in cancer progression and remodeling of the ectocervix [28]. Thus, MMP-2 and MMP-9 have potential use for the detection of cervical lesions and cancer. In this study, we found that PA dramatically inhibited migration and invasion of HeLa cells through downregulating MMP-2 and upregulating TIMP-2. This suggests that PA has potential as an anti-metastasis agent in the treatment of cervical cancer.

Mitogen-activated protein kinases (MAPKs) have roles in many biological functions and cellular responses, such as cell survival, proliferation, invasion, and apoptosis, depending on the stimulus intensity and duration, as well as the cell type. In the present study, we attempted to determine the role of several factors, including ERK1/2, p38, and JNK1/2, on the PA-induced downregulation of MMP-2, upregulation of TIMP-2, and inhibition of cell migration and invasion. Some studies show that Isothiocyanatesinhibited MMP-9 activity by inactivating FAK/ERK/AKT pathways in tumor cells [29]. Suppression of ERK/MMP-2 expression contributes to Silibinin-inhibited cell migration and invasion in human osteosarcoma MG-63 cells [30]. Fisetin could inhibit the migration and invasion of A549 cells through ERK1/2 inhibition and reduced the expressions of MMP-2 and u-PA [31]. In addition, acacetin suppressed TPA-induced cell invasion and MMP-9/uPA activities, and the suppression of JNK1/2 pathway in human lung cancer cells [32]. Naringenin possesses an anti-invasive effect against HCC cells via the inhibition of TPA-mediated MMP-9 expression, associated with inactivation of ERK1/2 and JNK1/2 pathways [33].Based on these observations, our results show that PA significantly suppressed the ERK1/2 signaling pathway, but not the JNK1/2 and p38 pathways. We also found that PD98059 (a specific MEK1/2 inhibitor) significantly enhanced the inhibitory effects of PA on cell migration and invasion, and also enhanced its downregulation of MMP-2 and its upregulation of TIMP-2. Furthermore, we observed that PA notably suppressed the effects of TPA on upregulation of ERK1/2 activity, upregulation of MMP-2 expression, and cell invasion and motility. These findings suggest that PA has strong in vitro anti-cancer activity against human cervical cancer cells

The present results suggest that the anticancer activity of PA is due to its suppression of cell proliferation, invasion, and motility by downregulating the ERK1/2 signaling pathway and MMP-2 expression, and upregulating TIMP-2 expression. Moreover, PA significantly inhibited the TPA-induced invasive motility of HeLa cells, the TPA-induced ERK1/2 activation, and the TPA-induced MMP-2 upregulation in HeLa cells. To our knowledge, this study is the first to demonstrate the effects and molecular mechanisms underlying the anti-cancer effects of PA against human cervical cancer cells in vitro.

## 4. Materials and Methods

### 4.1. Reagents 

PA was purchased from ChemFaces (Wuhan, China). A stock solution of Paeruptorin A (PA) was made at a concentration of 100 mM in dimethyl sulfoxide (DMSO) and stored at −20 °C. Antibodies against cyclin D1 (sc-717), p21 (sc-397), Skp2 (sc-7164), p27 (sc-528), TIMP-1 (sc-5538), TIMP-2 (sc-5539), p-ERK1/2 (sc-16982), ERK (sc-94), p-JNK (sc-6254), JNK (sc-571), p-p38 (sc-17852-R), p38 (sc-7972) and β-actin (sc-47778) were purchased from Santa Cruz Biotechnology (Santa Cruz, CA, USA). MMP-2 (ab92536) and MMP-9 (ab137867), HRP-conjugated anti-mouse (sc-516102) and anti-rabbit (sc-2004) secondary antibodies were purchased from Abcam (Cambridge, UK). The MEK1/2 inhibitor PD98059 was purchased from Calbiochem (San Diego, CA, USA). MTT was purchased from Sigma (St. Louis, MO, USA). All stock solutions were wrapped in foil and stored at −20 °C until use.

### 4.2. Cell Culture and Culture Condition

Human HeLa cervical cancer cells (BCRC No. 60005) was obtained from the Bioresources Collection and Research Center, Food Industry Research and Development Institute (Hsinchu, Taiwan). Human cervical adenocarcinoma SiHa cells (ATCC HTB35) were obtained from the American Type Culture Collection (Rockville, MD, USA). They were maintained in Dulbecco’s modified Eagle’s medium (DMEM, Gibco-Invitrogen Corporation, Carlsbad, CA, USA), supplemented with 10% fetal bovine serum (FBS, Gibco-Invitrogen Corporation) and 1% antibiotics (10,000 U/mL penicillin, 10 μg/mL streptomycin (Invitrogen Life Technologies, Carlsbad, CA, USA)) in a humidified atmosphere with 5% CO_2_ at 37 °C. To determine the effects of PA on activation of MAPKs expression, HeLa cells were treated with different concentration of PA (10, 20 and 30 μM) for 24 h under serum medium. The effects of PA on TPA induced phosphorylation of MAPKs expression, the HeLa cells were pretreated with PA (20 μM) for 2 h and then treated with or without TPA (50 ng/mL) for different times (0, 30, 60, 90 and 120 min) under serum free medium. The PA-inhibited TPA-induced migration, invasion, phosphorylation of ERK1/2 and MMP-2 expression in HeLa cellswere examined by pretreating the HeLa cells with the different concentration of PA (20 and 30 μM) for 2 h before treated with or without TPA (50 ng/mL)for 24 h under serum free medium. The protein levels of p-ERK1/2, ERK1/2 and MMP-2 was determined by western blotting, the mRNA levels of MMP-2 was measured by RT-qPCR analysis.

### 4.3. Immunoblotting

To isolate total proteins, cells were washed with cold PBS and resuspended in lysis buffer (50 mM Tris, pH 7.5, 0.5 M NaCl, 1.0 mM Ethylenediaminetetraacetic acid (EDTA), 10% glycerol, 1 mM β-mercaptoethanol (β-ME), 1% Nonidet P-40 (NP40) with mixtures of proteinase inhibitors and phosphatase inhibitors (Roche Molecular Biochemicals, Pleasanton, CA. USA). After incubation for 30 min on ice, the supernatant was collected by centrifugation at 12,000× *g* for 15 min at 4 °C, and the protein concentration was determined by the Bradford method. Samples containing equal amounts of total protein (40 µg) were separated by 12% SDS-PAGE, and transferred onto PVDF membrane (Life Technologies, Carlsbad, CA, USA). The membranes were blocked with a buffer (5% non-fat dry milk, 20 mM Tris-HCl, pH 7.6, 150 mMNaCl, 0.1% Tween 20) for at least 1 h at room temperature and then incubated with primary antibodies in the above solution on an orbital shaker at 4 °C overnight. Then, the membranes were incubated with horseradish peroxidase-linked secondary antibodies (anti-rabbit, anti-mouse, or anti-goat IgG). Antibody-bound protein bands were detected using highly sensitive Immobilon Western Chemiluminescent HRP Substrate (Millipore, Billerica, MA, USA), and photographed using the Luminescent Image Analyzer LAS-4000mini.

### 4.4. Cell Viability Assay

To determine the effect of PA on HeLa and SiHa cell viability, cells were subjected to the MTT assay. The absorbance of blue formazan, which is directly proportional to the number of viable cells, was measured at 570 nm, with correction for background, using an enzyme-linked immunosorbent assay plate reader.

### 4.5. Colony Formation Assay 

HeLa and SiHa cells were seeded into 6-well plates for 2 weeks. Colonies with more than 50 cells were stained with 0.5% crystal violet for 30 min at room temperature. Three independent experiments were performed.

### 4.6. Flow Cytometric Analysis

Cells were centrifuged at 800 rpm at 4 °C for 5 min, washed with ice-cold PBS, and stained with propidium iodide (PI) buffer (4 μg/mL PI, 1% Triton X-100, 0.5 mg/mL RNase A in PBS). The DNA content using a Muse Cell Analyzer flow cytometry and analysis data by the Muse^®^ Cell Analyzer Assays (Millipore, Darmstadt, Germany). Cells were gated to exclude cell debris, doublets, and clumps. The apoptotic cells with hypodiploid DNA content were detected in the sub-G1 region. 

### 4.7. Migration and Invasion Assay

HeLa and SiHa cells were treated with different concentrations of PA in the absence or presence of PMA (50 ng/mL) for 24 h by using 48-well modified Boyden chambers containing membrane filter inserts with 8-μm pores (Corning Incorporated Life Sciences, Tewksbury, MA, USA). These filter inserts were coated with Matrigel (BD Biosciences, Billerica, MA, USA) prior to the invasion assay. The lower compartment was filled with DMEM containing 10% FCS. Cells were placed in the upper part of the chamber, which contained serum-free medium, and incubated for 16~24 h. Cell migration and invasion were determined by counting cells that migrated to the lower side of the filter at 100× and 200× magnification, respectively. Four microscopic fields were counted for each filter, and each sample was assayed in triplicate.

### 4.8. Wound Healing Assay

HeLa cells (4 × 10^5^ cells/well) were seeded in a 6-well plate and grown overnight to 90% confluence. After removing the medium, the cell monolayer was scratched with a 200-μL pipette tip to create a wound. The cells were then washed twice with PBS to remove floating cells and fresh medium (contain 2 μg/mL of mitomycin c) was added. Cells migrating from the leading edge of the wound were photographed at 0 and 24 h.

### 4.9. Reverse Transcription and Real-Time PCR Assay

Total RNA was isolated from cultured cells by homogenization in TRIzol reagent (Invitrogen, Carlsbad, CA, USA) was used for RNA extraction. Standard reverse transcription and real-time PCR protocols were used. For reverse transcription, the samples were incubated at 25 °C for 10 min, real-time PCR was initiated with a hot start (10 min at 95 °C, 1 cycle), and samples were then subjected to 40 cycles at 95 °C for 15 s and 60 °C for 1 min. Data were analysed using the StepOne real-time PCR system (Applied Biosystems, Foster City, CA, USA). The primers were as follows: MMP-2 forward primer 5′-TGGCAAGTACGGCTTCTGTC-3′, MMP-2 reverse primer 5′-TTCTTGTCGCGGTCGTAGTC-3′; MMP-9 forward primer 5′-ACGACGTCTTCCAGTACCGA-3′; MMP-9 reverse primer 5′-TCATAGGTCACGTAGCCCAC-3′; TIMP-1 forward primer 5′-AGAGTGTCTGCGGATACTTCC-3′, TIMP-1 reverse primer 5′-CCAACAGTGTAGGTCTTGGTG-3′; TIMP-2 forward primer 5′-AAGCGGTCAGTGAGAAGGAAG-3′, TIMP-2 reverse primer 5′-GGGGCCGTGTAGATAAACTCTAT-3′; Glyceraldehyde 3-phosphate dehydrogenase (GAPDH) forward primer 5′-CATCATCCCTGCCTCTACTG-3′; GAPDH reverse primer 5′-GCCTGCTTCACCACCTTC-3′ (MISSION BIOTECH, Taipei, Taiwan). Relative gene expression was obtained after normalization with endogenous GAPDH and determination of the difference in threshold cycle (*C*t) between treated and untreated cells using 2^−ΔΔ*C*t^ method.

### 4.10. Statistical Analysis

Each experiment was performed at least three times. Results are presented as means ± standard errors, and statistical comparisons were made using Student’s *t*-test. Significance was defined as a *p*-value below 0.05 or 0.01.
